# Characterization of tumor endothelial cells (TEC) in gastric cancer and development of a TEC-based risk signature using single-cell RNA-seq and bulk RNA-seq data

**DOI:** 10.18632/aging.205928

**Published:** 2024-06-12

**Authors:** Meng Fan, Xiaofei Xu, Yu Hu

**Affiliations:** 1 Department of Gastrointestinal Surgery, Zhu Cheng People’s Hospital, Weifang, China

**Keywords:** tumor endothelial cells, gastric cancer, risk signature, immunotherapy

## Abstract

Background: Tumor endothelial cells (TECs) are essential participants in tumorigenesis. This study is focused on elucidating the TEC traits in gastric cancer (GC) and constructing a prognostic risk model to predict the clinical outcome of GC patients.

Methods: Single-cell RNA sequencing (scRNA-seq) data were obtained from the GEO database. Using specific markers, the Seurat R package aided in processing scRNA-seq data and identifying TEC clusters. Based on TEC cluster-associated genes identified by Pearson correlation analysis, TEC-related prognostic genes were screened by lasso-Cox regression analysis, thereby constructing a risk signature. A nomogram was created by combining the risk signature with clinicopathological features.

Results: Based on the scRNA-seq data, 5 TEC clusters were discovered in GC, with 3 of them showing prognostic associations in GC. A total of 163 genes were pinpointed among 3302 DEGs as significantly linked to TEC clusters, leading to the formulation of a risk signature comprising 8 genes. Furthermore, there was a notable correlation between the risk signature and the immune cell infiltration. Multivariate analysis findings indicated that the risk signature served as an independent prognostic factor for GC. Moreover, its efficacy in forecasting immune response was validated.

Conclusion: TEC-based risk model is highly effective in predicting the survival outcomes of GC patients and can forecast the immune response. Targeting TECs may significantly inhibit tumor progression and enhance the efficacy of immunotherapy.

## INTRODUCTION

Gastric cancer (GC) is a common worldwide cancer that presents a major risk to health [1, 2]. Its intricate evolution involves the interplay of dietary factors, host genes, Helicobacter pylori infection, and environmental elements [3–5]. Because of atypical symptoms, gastric cancer at an early stage is frequently not identified until it has progressed to a more advanced stage. This leads to a poor prognosis characterized by a high likelihood of local recurrence and distant metastasis [6, 7].

Tumor endothelial cells (TECs) are crucial components of the tumor microenvironment (TME) and play a pivotal role in promoting tumorigenesis and metastasis [8–13]. Throughout tumor development, TECs contribute not only to the formation of new blood vessels but also impact the biological behavior of tumor cells by secreting a variety of bioactive molecules, including growth factors, cytokines, and enzymes [12, 14, 15]. Additionally, TECs possess the capability to modulate immune responses. They can produce immunosuppressive molecules such as TGF-β and IL-10, which dampen the activity of immune cells, thereby facilitating tumor cells to evade immune detection and destruction [14].

Additionally, TECs are also able to influence the invasive and metastatic ability of tumor cells through direct interaction with them [16]. For instance, by secreting enzymes like matrix metalloproteinases (MMPs) that aid in tumor cell extravasation and basement membrane penetration, TECs assist tumor cells in traversing the vessel wall, entering the bloodstream, and establishing distant metastases [14, 17–19]. Of note, TEC has the capacity to interact with a variety of cells and molecules present in the TME [14]. Other cell types within the tumor microenvironment (TME), such as cancer-associated fibroblasts (CAFs) and immune cells, synergistically cooperate with TEC in establishing a favorable microenvironment for tumor progression and metastasis by secreting signaling molecules or engaging in direct cell-to-cell interactions [20, 21].

It has been reported that distinguished from normal endothelial cells (NECs), TECs exhibit chromosomal instability, altered gene expression, enhanced proliferative capacities, and resistance to anti-angiogenic drugs [22, 23]. Targeting TECs can inhibit tumor progression and prolong patient survival by blocking the formation of tumor blood vessels [16, 22, 24]. Based on the scRNA-seq analysis, the study by Yin et al. revealed a TEC cluster in GC tissues that exclusively expressed IGFBP5 and displayed malignant characteristics [23]. Patients with TECs overexpressing IGFBP5 and IGFBP3 demonstrated significantly lower overall survival (OS). While numerous studies have been undertaken, a comprehensive understanding of the systematic characteristics of TECs and their correlation with GC prognosis and response to immunotherapy remains elusive.

In this research, we acquired scRNA-seq and transcriptome data, identifying distinct TEC clusters and developing a risk signature based on TECs. We assessed the clinical significance of the risk signature and examined the relationship between the risk signature and TME, immune response. Subsequently, we created a new nomogram that combines the risk signature with clinicopathological characteristics, to enhance its performance in predicting the clinical outcome of GC. Our study provides new insights into the molecular mechanisms underlying the occurrence and progression of GC, allowing for more individualized therapies.

## METHODS

### Data acquisition and processing

From the public online website (https://dna-discovery.stanford.edu/research/datasets/) [25], we acquired scRNA-seq data of GC tissues, which included 9 GC samples, 10 normal samples, 2 samples of peripheral blood mononuclear cells (PBMC), and 1 sample of metaplasia. Our focus was specifically on the 9 GC tissue samples and their corresponding normal samples.

We used strict standards for scRNA-seq data, ensuring that each gene was expressed in a minimum of 3 cells and that each cell expressed at least 250 genes. To ensure quality control (QC) with the Seurat R package [26], cells with mitochondrial gene expression percentages higher than 15% were eliminated. Additional filtering based on QC criteria (nFeature RNA ≥ 200 and nFeature RNA ≤ 5000 and nCount_RNA ≥ 500) was applied. Normalization was executed using Seurat R package’s “NormalizeData” function, resulting in 37,440 cells. We adjusted the number of principal components (PCs) to 30 for the generation of cell clusters. The identification of cell clusters was performed using the FindClusters function with a resolution set to 1, and the results were visualized through UMAP method.

The Cancer Genome Atlas (TCGA) provided RNA-seq data, single-nucleotide variant (SNV), copy number variant (CNV) data, and clinical information for GC. After additional curation, the transcriptome data yielded a total of 345 tumor samples and 32 para-cancerous samples. Furthermore, for further validation, GSE62254 (300 GC samples) and GSE15459 (192 GC samples) cohorts were obtained from the GEO database (https://www.ncbi.nlm.nih.gov/geo/). Moreover, ten pathways related to cancer were acquired from the literature [27].

### Definition of TEC

The Seurat R package enabled a thorough re-examination of scRNA-seq data to characterize the signature of TECs. The function ‘FindIntegrationAnchors’ was used to handle batch effects in 18 samples. The uniform manifold approximation and projection method with 30 principal components (PCs) and a resolution of 0.25 was employed for non-linear dimensional reduction. The process of clustering was executed by utilizing the functions ‘FindNeighbors’ and ‘FindClusters’. The function ‘RunUMAP’ was utilized to apply UMAP dimensionality reduction. Endothelial cells were characterized by 8 marker genes, namely VWF, ENG, PLVAP, MCAM, PECAM1, CLDN5, SELE, and SELP. Furthermore, UMAP dimensionality reduction was applied to the endothelial cell clusters. Marker genes for each TEC cluster were determined using the FindAllMarkers function. The Kyoto Encyclopedia of Genes and Genomes (KEGG) enrichment analysis on TEC cluster marker genes was conducted using the clusterProfiler R package [28]. Additionally, the CNV characteristics within the TEC clusters were analyzed using the Copykat R package to discern between malignant and non-malignant cells in each sample [29].

### TEC-related gene identification

The limma R package was used to identify DEGs between tumor and normal tissue, with an adjusted *p*-value of less than 0.05 and an absolute log2 (fold change) greater than 1 [30]. Correlations between DEGs and TEC clusters were evaluated, with a focus on the identification of crucial TEC-associated genes. The genes associated with prognosis were further determined through univariate Cox regression analysis in the survival package with a significance level of *p* < 0.05. In order to reduce the number of genes, a lasso-Cox regression analysis was conducted, followed by a multivariate Cox regression analysis using a stepwise regression approach. Based on the outcomes of the multivariate Cox model, a risk signature was developed utilizing the formula: risk score = Σβi × Expi, where i represents the gene in the risk signature, expi denotes the expression of gene i, and βi indicates the coefficients of gene i in the multivariate Cox model. Subsequent to zero-mean normalization, the patients were stratified into high- and low-risk groups. The predictive performance of the risk signature was evaluated through receiver operating characteristic curve (ROC) analysis using the timeROC R package.

### Immune landscape analysis

The CIBERSORT algorithm evaluated the proportions of 22 distinct immune cell types within the context of GC [31]. By computing StromalScores, ImmuneScores, and ESTIMATEScores, the ESTIMATE algorithm facilitated the investigation of the connection between the risk genes and the TME [32].

### The ability to respond to immune checkpoint inhibitors

We acquired transcriptomic and clinical information from GC patients who received treatment with immunotherapy from the IMvigor210 and GSE78220 cohorts [33, 34]. The assessment was conducted to determine the potential significance of the risk signature in predicting the effectiveness of immune checkpoint inhibitors (ICIs).

### Statistical analysis

R software (Version 4.2.1) was utilized for all the analyses. A *p*-value less than 0.05 was deemed to be statistically significant.

### Data availability statement

The datasets presented in this study can be found in online repositories. The names of the repository/repositories and accession number(s) can be found in the article.

## RESULTS

### Screening of TECs in scRNA-seq samples

**Figure 1:**
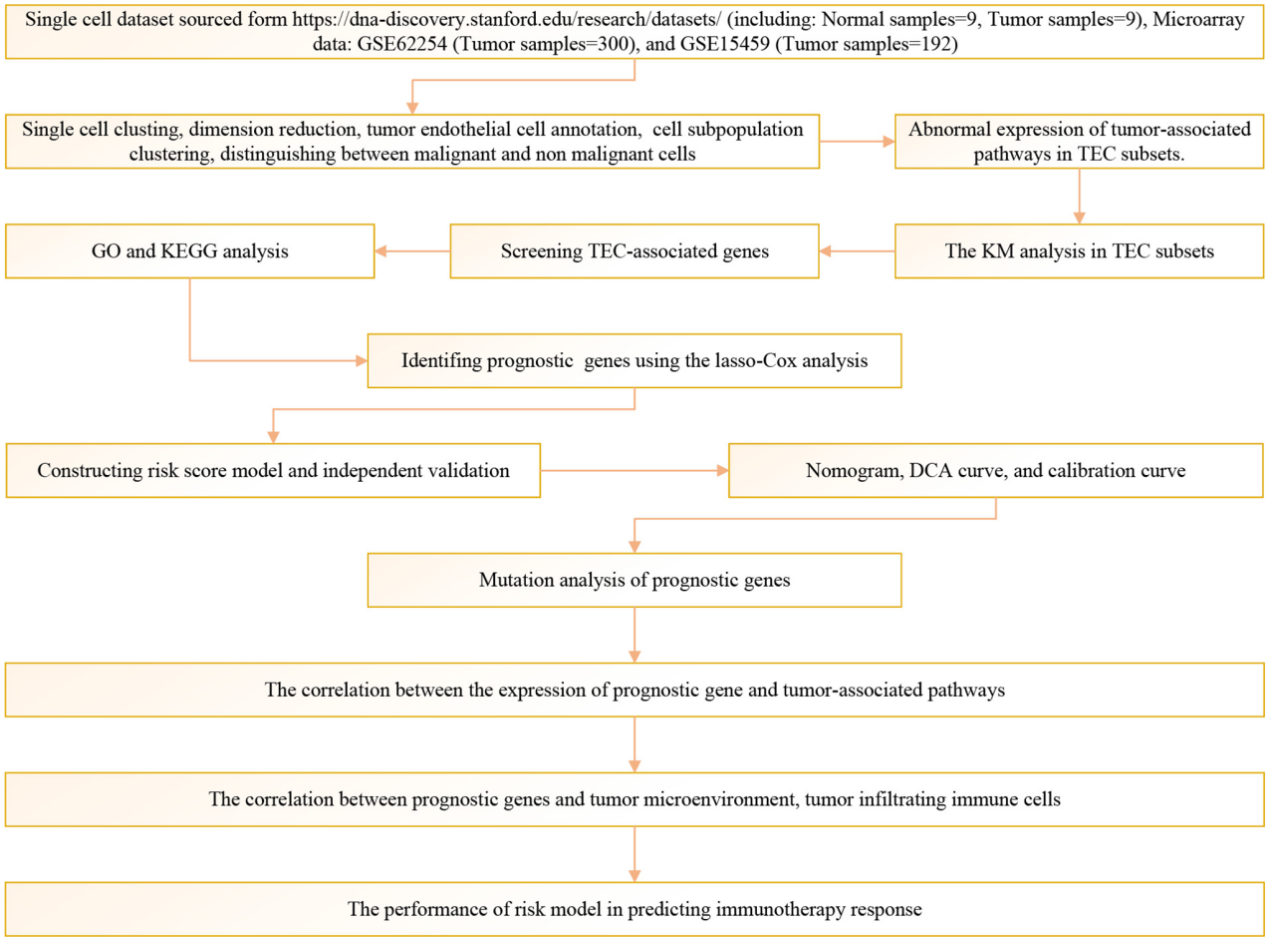
The flow chart of this study.

**Figure 2:**
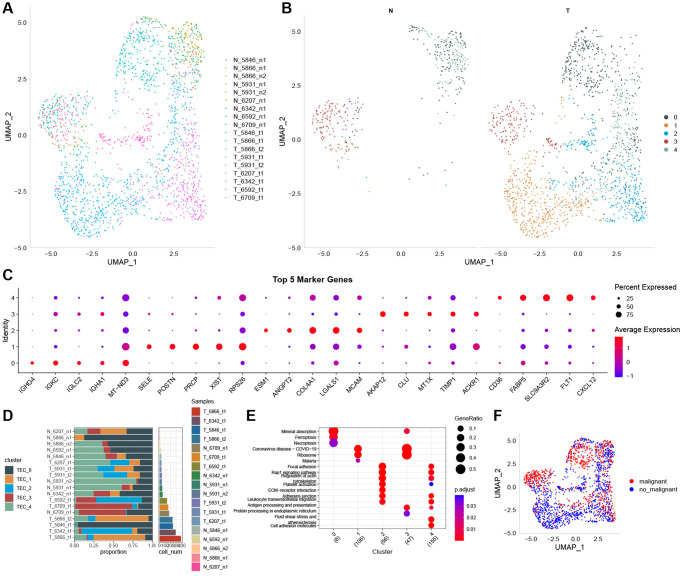
**The identification of TEC clusters based on scRNA seq data of GC patients.** (**A**) UMAP plot of the distribution of 18 samples. (**B**) UMAP plot of the distribution of five TEC clusters after clustering. (**C**) Dot plot of the top 5 marker gene expression of TEC clusters. (**D**) The proportion of the five TEC clusters in tumor samples and normal samples. (**E**) KEGG enrichment analysis of 5 TEC clusters. (**F**) UMAP distribution map of malignant and non-malignant cells predicted by Copykat package.

Figure 1 depicts the study’s flow chart. At first, a total of 37,440 cells were obtained from scRNA-seq data after an initial QC. After log-normalization and dimensionality reduction, a total of 36 subgroups were observed, uncovering a single TEC population distinguished by 8 marker genes: VWF, ENG, PLAVP, MCAM, PECAM1, CLDN5, SELE, and SELP (Supplementary Figure 1A, 1B). Afterward, TEC group cells went through further clustering and dimensionality reduction. The TEC populations were divided into five clusters using the identical clustering algorithm (Supplementary Figure 1C, 1D). In Figure 2A, the UAMP plot shows the distribution of 18 samples. As a result, five TEC clusters were finally generated and used for subsequent analysis (Figure 2B). 601 DEGs were identified among the 5 TEC clusters. Figure 2C displays the expression of the top 5 DEGs, which act as marker genes for the TEC clusters. Figure 2D indicates the proportion of the five TEC clusters in each sample. The analysis of KEGG showed enrichment in different pathways, including mineral absorption, ferroptosis, necroptosis, ribosome, focal adhesion, rap 1 signaling pathway, regulation of actin cytoskeleton, platelet activation, ECM-receptor interaction, adherens junction, and cell adhesion molecules (Figure 2E). Moreover, five TEC clusters were identified to comprise 839 tumor cells and 1137 normal cells according to CNV traits (Figure 2F).

### Expression of cancer-related pathways in TEC

**Figure 3:**
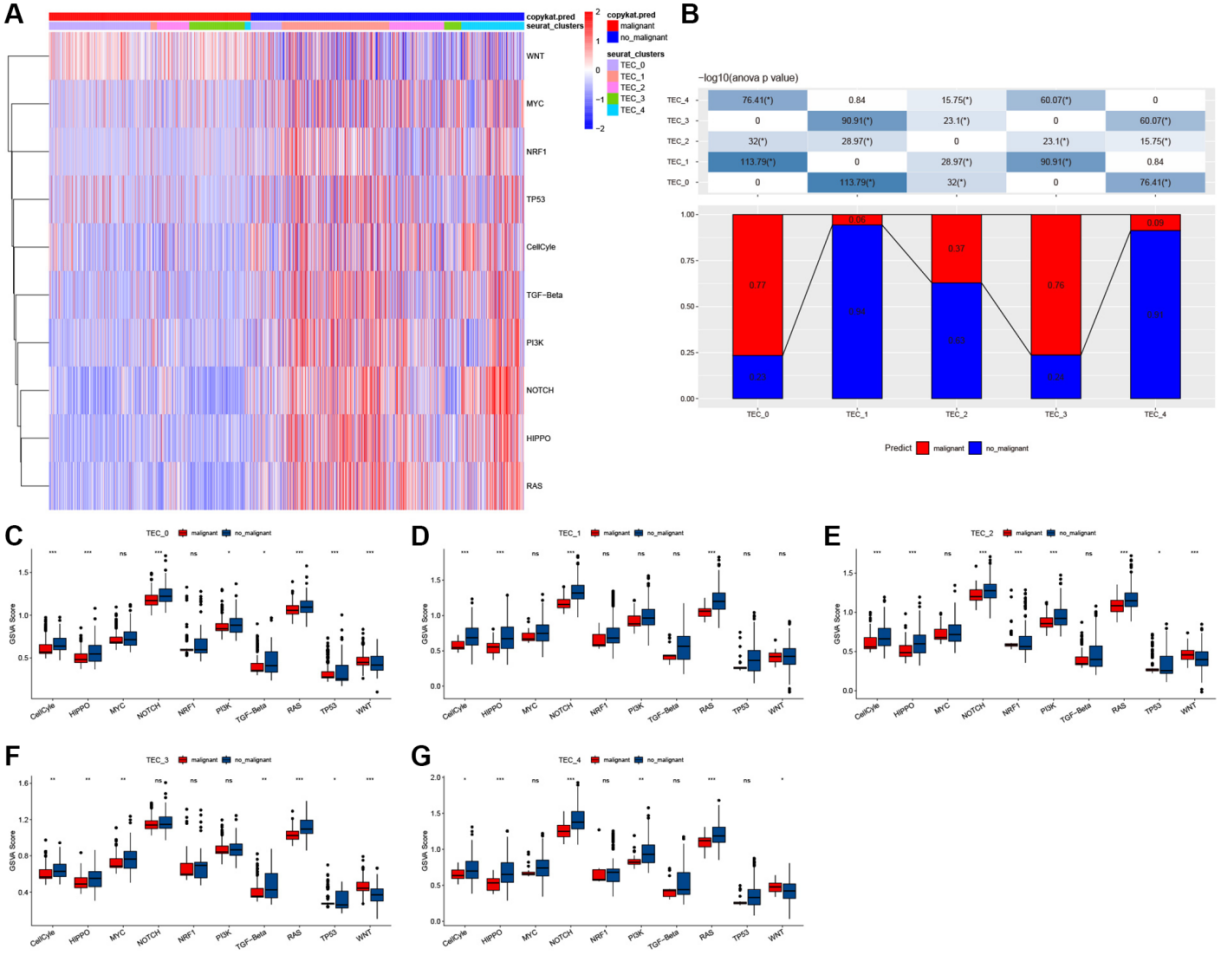
**The characteristics of tumor-related pathways in TEC clusters.** (**A**) Heatmap of 10 tumor-related pathway scores enriched in TEC cells. (**B**) Comparison of TEC clusters in malignant and non-malignant cells. (**C**–**G**) Comparison of GSVA score of each pathway between malignant and non-malignant cells in TEC_0 (**C**), TEC_1 (**D**), TEC_2 (**E**), TEC_3 cluster (**F**), and TEC_4 cluster (**G**). ^*^P < 0.05, ^**^P < 0.01, ^***^P < 0.001, ^****^P < 0.0001, Abbreviation: ns: not significant.

To explore the relationship between TECs and tumorigenesis, we calculated GSVA scores for ten oncogenic signaling pathways across distinct TEC subtypes, utilizing the GSVA R package. The heatmap indicated the significant activation of multiple oncogenic signaling pathways in TEC_1 and TEC_4 (Figure 3A). Notably, the TEC_0 and TEC_3 clusters exhibit a substantially higher proportion of malignant cells compared to the other three clusters (Figure 3B). Furthermore, we conducted a comprehensive comparison of GSVA scores for oncogenic signaling pathways among five TEC clusters, revealing distinctions between malignant and non-malignant cells. The findings suggest that the cell cycle, HIPPO, NOTCH, and RAS pathways are highly activated in non-malignant cells (Figure 3C–3G).

**Figure 4:**
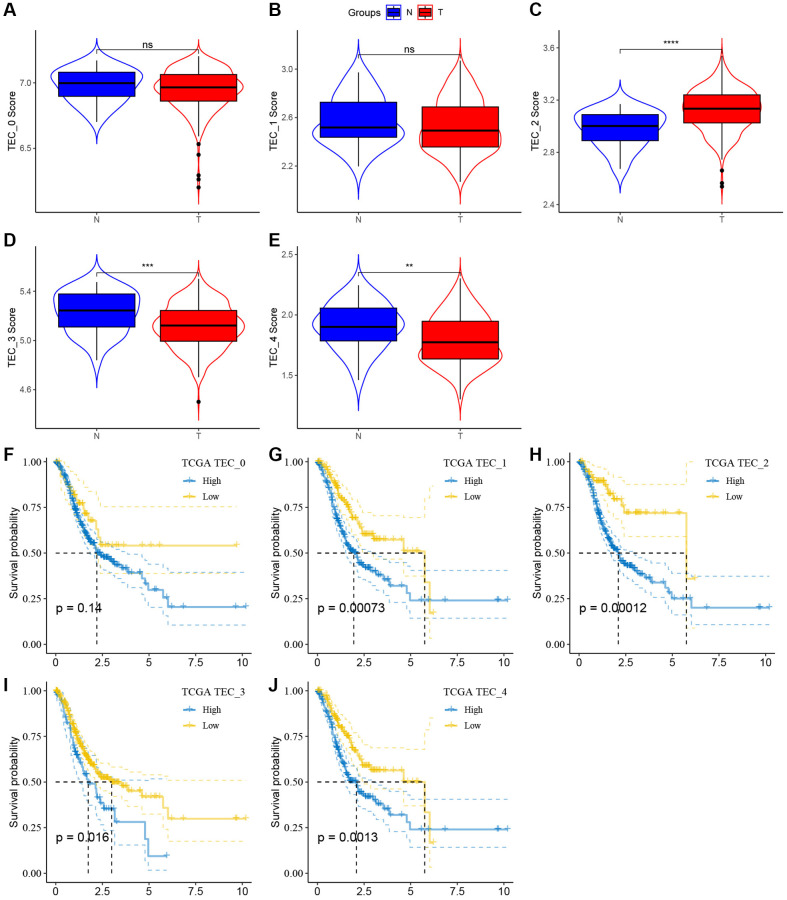
**The associations between the five TEC cluster and prognosis of GC patients.** (**A**–**E**) Comparison of five TEC scores in cancer and normal tissues, ^**^P < 0.01, ^***^P < 0.001, ^****^P < 0.0001, Abbreviation: ns: not significant. (**F**–**J**) K-M curves of the high and low TEC score groups in the TEC_0 cluster (**F**), TEC_1 cluster (**G**), TEC_2 cluster (**H**), TEC_3 cluster (**I**), and TEC_4 (**J**).

In order to evaluate the correlation between TEC clusters and prognosis, the ssGSEA scores of the marker genes (the top 5 DEGs of TEC clusters) were calculated for each TEC cluster using the TCGA cohort. The findings showed elevated TEC scores for the TEC_2 cluster in tumor tissues compared to normal tissues, whereas the TEC_3 and TEC_4 clusters displayed a contrasting pattern (Figure 4A–4E). Kaplan-Meier (KM) survival analysis showed that, in the high TEC score group, GC patients exhibited a worse prognosis across TEC_1, TEC_2, TEC_3, and TEC_4 clusters compared to the low-TEC score group. Conversely, TEC_0 showed no significant association with GC prognosis (Figure 4F–4J). Supplementary Figure 2 revealed that the difference in TEC scores between different clinical variables such as T stage, N stage, M stage, and pathological stage.

### Discovering hub genes related to TEC

**Figure 5:**
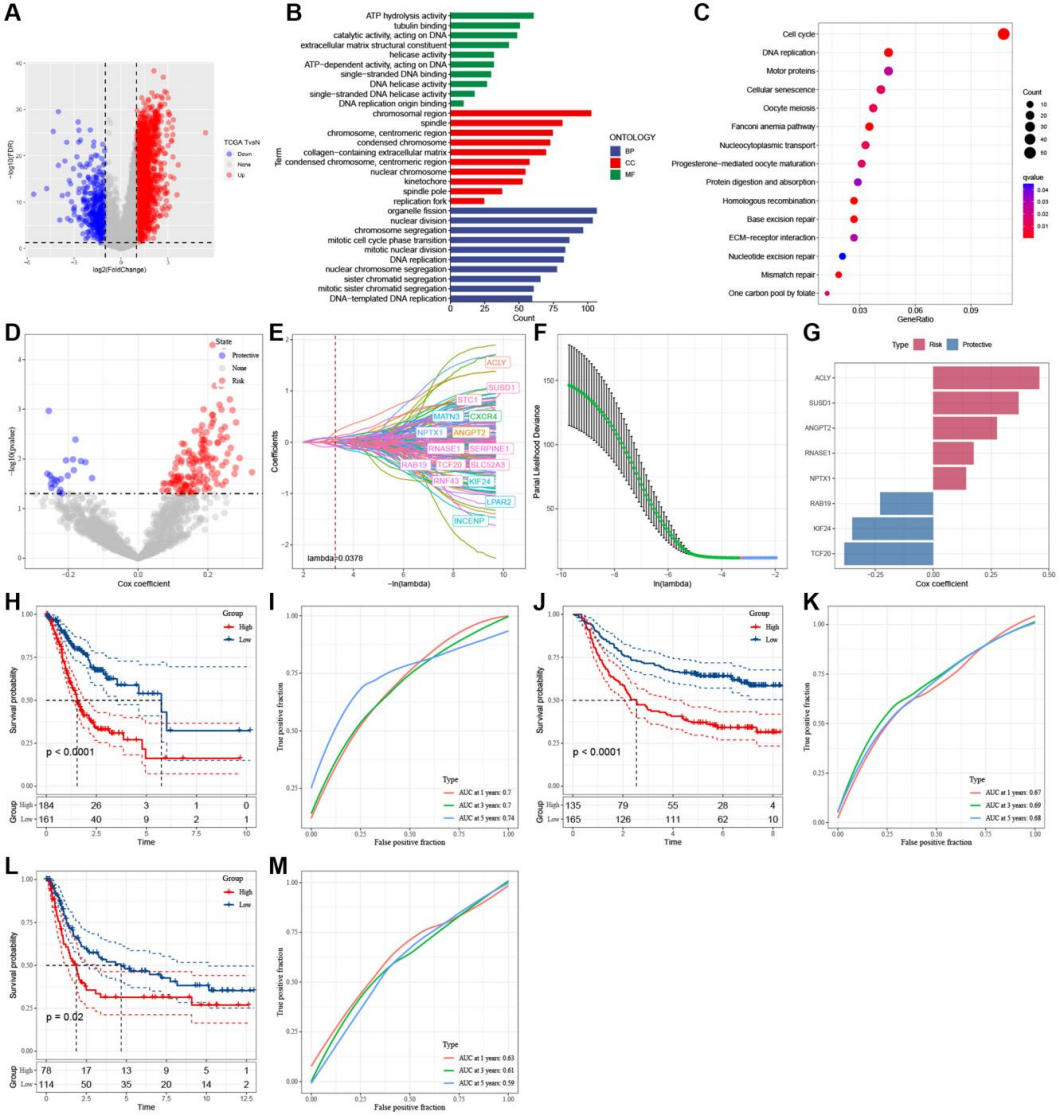
**Identification of the hub genes to construct a risk signature.** (**A**) Volcano plot of differentially expressed genes of cancer and normal tissues in TCGA cohort. (**B**) GO analysis. (**C**) KEGG analysis. (**D**) Volcano plot of prognosis-related genes identified from univariate Cox regression analysis. (**E**) The trajectory of each independent variable with lambda. (**F**) Plots of the produced coefficient distributions for the logarithmic (lambda) series for parameter selection (lambda). (**G**) The multivariate Cox coefficients for each gene in the risk signature. (**H**) K-M curves of risk model constructed by 8 genes in TCGA cohort. (**I**) ROC curves of risk model constructed by 8 genes in TCGA cohort. (**J**) K-M curves of risk model constructed by 8 genes in GSE62254 cohort. (**K**) ROC curves of risk model constructed by 8 genes in GSE62254 cohort. (**L**) K-M curves of risk model constructed by 8 genes in GSE15459 cohort. (**M**) ROC curves of risk model constructed by 8 genes in GSE15459 cohort.

To construct a risk signature, we conducted a differential analysis between tumor and normal tissues and identified a total of 2717 DEGs, comprising 2259 genes with up-regulation and 458 genes with down-regulation, as illustrated in Figure 5A. Regarding biological processes (BP), these genes exhibited enrichment in organelle fission, nuclear division, chromosome segregation, DNA replication, mitotic nuclear division, and mitotic cell cycle phase transition (Figure 5B). Furthermore, KEGG analysis indicated that these genes are predicted to positively regulate the cell cycle, Fanconi anemia pathway, DNA replication, motor proteins, and cellular senescence (Figure 5C).

A total of 163 genes with predictive performance were identified through univariate Cox regression analysis (Figure 5D). The utilization of lasso-Cox regression analysis led to an additional decrease in the number of genes to 16, as illustrated in Figure 5E, 5F. Following a multivariate Cox regression analysis employing a stepwise regression approach, 8 genes were selected for incorporation into the risk signature (Figure 5G). These genes are ATP citrate lyase (ACLY), Sushi Domain Containing 1 (SUSD1), Angiopoietin 2 (ANGPT2), Ribonuclease A Family Member 1 (RNASE1), Neuronal Pentraxin 1 (NPTX1), RAB19, Kinesin Family Member 24 (KIF24), and Transcription Factor 20 (TCF20). The formula for calculating the risk score is as follows: RiskScore = 0.276 × ANGPT2 − 0.349 × KIF24 + 0.175 × RNASE1 + 0.459 × ACLY + 0.143 × NPTX1 − 0.229 × RAB19 + 0.369 × SUSD1 − 0.384 × TCF20. GC samples were categorized into high- and low-risk groups based on the median risk scores. Figure 5I, 5K, 5M show that the AUC values of the risk model varied between 0.7, 0.7, and 0.74 in the TCGA cohort, 0.67, 0.69, and 0.68 in the GSE62254 cohort, and 0.63, 0.61, and 0.59 in the GSE15459 cohort for 1-, 3-, and 5-year survival. In the TCGA and GEO cohorts, the KM survival analyses revealed that high-risk patients had worse clinical outcomes than low-risk patients, as shown in Figure 5H, 5J, 5L.

### Identification of independent risk factors and nomogram development

**Figure 6:**
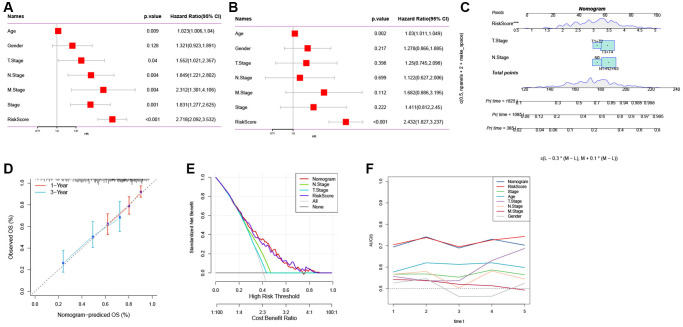
**The development of a nomogram for predicting the prognosis of GC.** (**A**, **B**) Univariate and multivariate Cox analysis of risk score and clinicopathological characteristics. (**C**) Nomogram model integrating the risk score and T stage, N stage was constructed. (**D**) Calibration curves for 1, 3 years of nomogram. (**E**) Decision curve for nomogram. (**F**) Comparison of predictive capacity of clinicopathological features and the nomogram using time-ROC analysis. ^***^P < 0.001.

To enhance the predictive accuracy of the risk model, we integrated clinical pathological features and risk scores and conducted univariate and multivariate Cox regression analyses. The results confirmed that the risk score was the most significant independent prognostic factor for GC (Figure 6A, 6B). Additionally, based on the risk score and clinical variables such as T stage and N stage, we constructed a nomogram to assess the clinical outcomes of GC patients (Figure 6C). The calibration curve demonstrated that the nomogram can effectively forecast the actual clinical outcomes (Figure 6D). As shown in Figure 6E, decision curve analysis (DCA) confirmed that the nomogram and risk score had better predictive efficacy in identifying high-risk patients compared to the N stage and T stage. Time-dependent ROC analysis demonstrated that, in the TCGA cohort, the area under the curve (AUC) of the risk score and nomogram was higher than other indicators (Figure 6F).

### Mutation and pathway analysis of the hub genes

**Figure 7:**
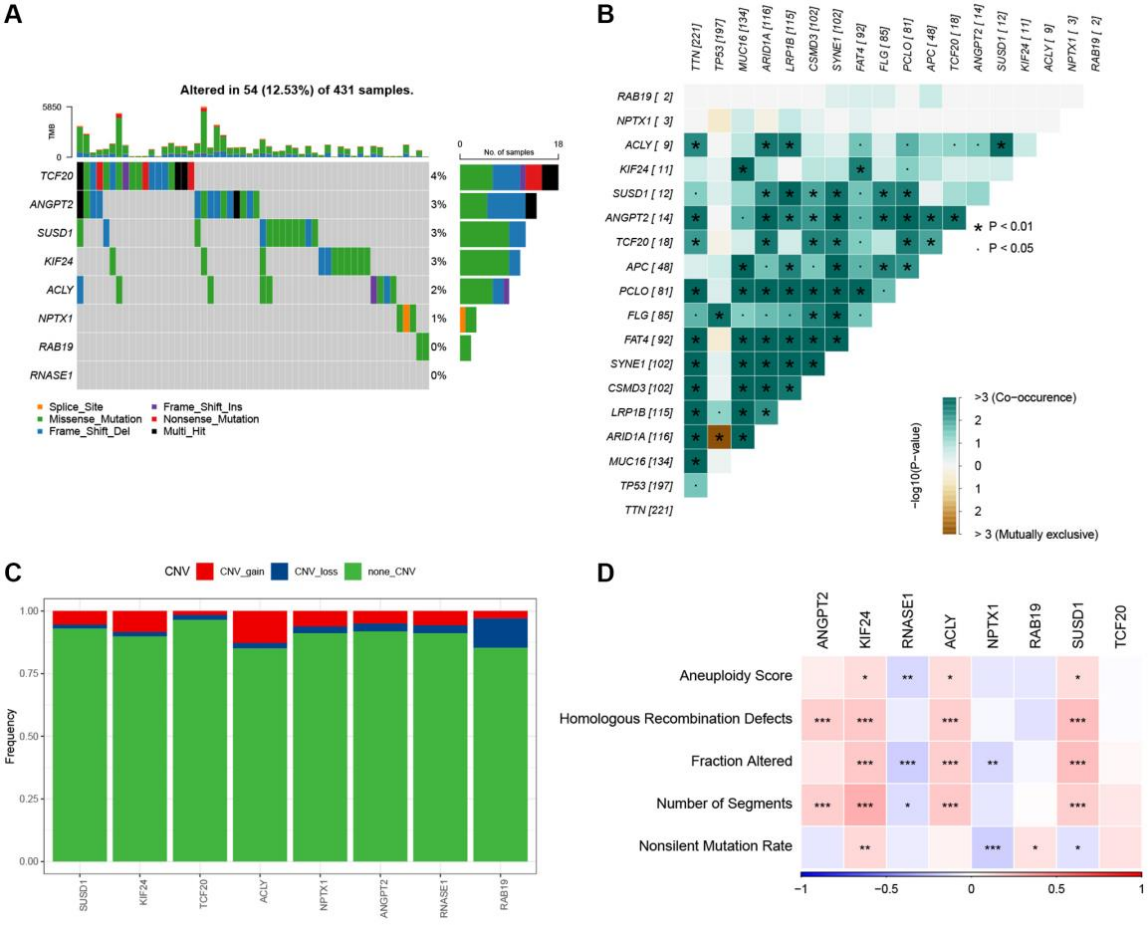
**The characteristics of mutations of the genes included in the risk signature.** (**A**) Waterfall diagram of SNV mutations of 8 key genes. (**B**) Colinearity and mutual exclusion analysis of 8 key genes and the 10 most mutated genes in tumors. (**C**) CNV mutations (gain, loss, none) of 8 key genes. (**D**) Correlation heatmap of 8 key genes with Aneuploidy Score, Homologous Recombination Defects, Fraction Altered, Number of Segments, and Nonsilent Mutation Rate.

**Figure 8:**
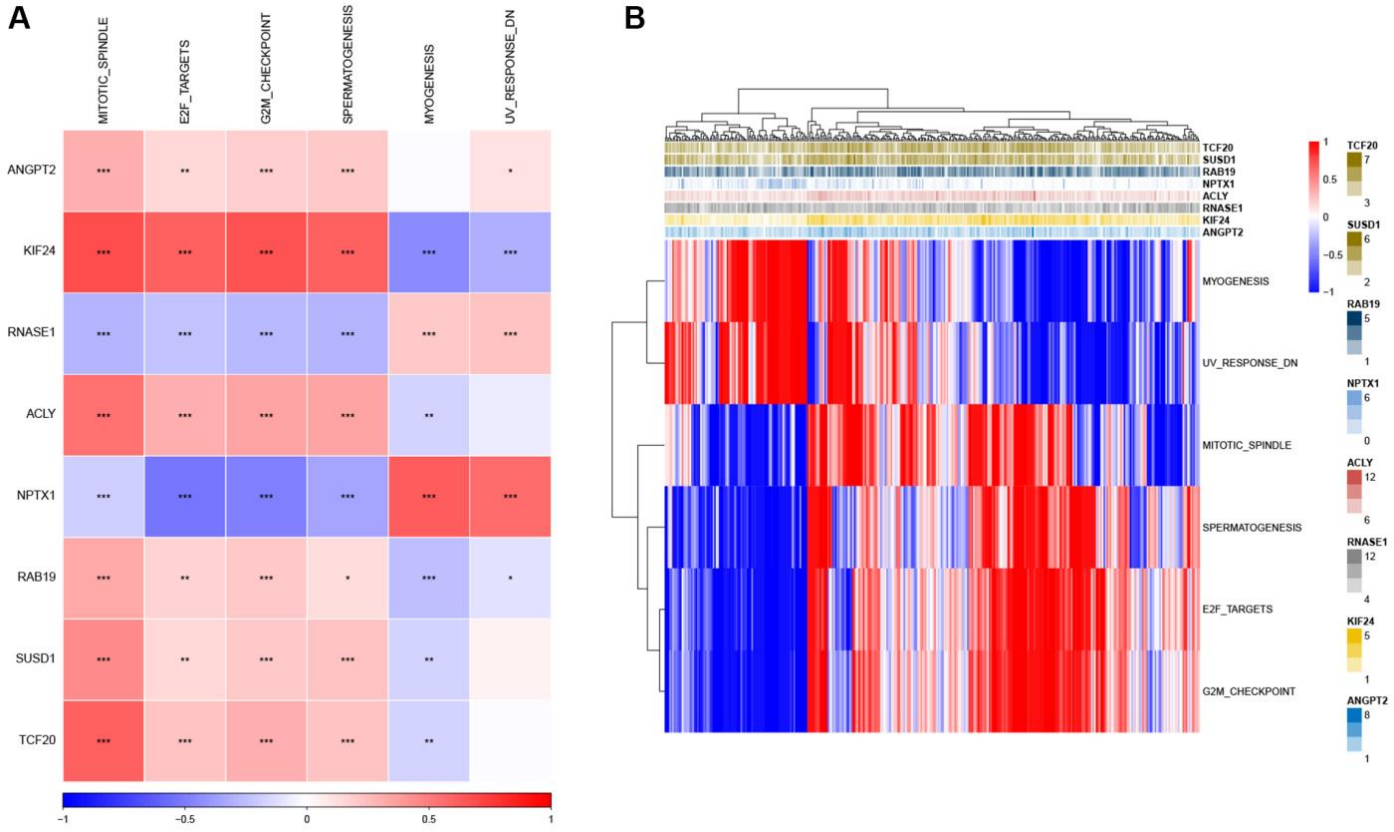
**Identification of the pathways in which the risk genes are implicated.** (**A**) Heatmap showing the correlation between genes and pathways. (**B**) Heatmap displaying the enrichment scores for key pathways. ^*^P < 0.05, ^**^P < 0.01, ^***^P < 0.001.

Next, we analyzed the SNV mutation status of 8 genes in the risk model. The results showed that TCF20, ANGPT2, SUSD1, KIF24, ACLY, and NPTX1 exhibited SNV mutations in GC samples, while no SNV mutations were found in RAB19 and RNASE1 (Figure 7A). The probability of co-occurrence among the identified key genes and the top 10 most mutated genes was examined. Figure 7B shows no significant co-occurrence probability between mutations in RAB19 and NPTX1. However, ACLY, SUSD1, ANGPT2, and TCF20 exhibit a notable probability of co-occurrence with mutations in TTN, ARID1A, FAT4, and PCLO. Among the 8 genes, that only a minimal number of GC samples experienced gain/loss of copy number variation (CNV) (Figure 7C). To further elucidate the associations between the risk genes and GC, we analyzed the correlations between these genes and several molecular signatures of GC. The results demonstrated that RNASE1 had significantly negative correlations with aneuploidy score, fraction altered, and number of segments, whereas ANGPT2, KIF24, ACLY, and SUSD1 showed significantly positive correlations with homologous recombination defects, fraction altered, and number of segments (Figure 7D). Furthermore, the analysis of pathways indicated a strong correlation between eight genes and six pathways, including myogenesis, UV-response-DN, mitotic spindle, spermatogenesis, E2F targets, and G2M checkpoints (Figure 8A, 8B).

### Associations between risk genes and tumor immunity

**Figure 9:**
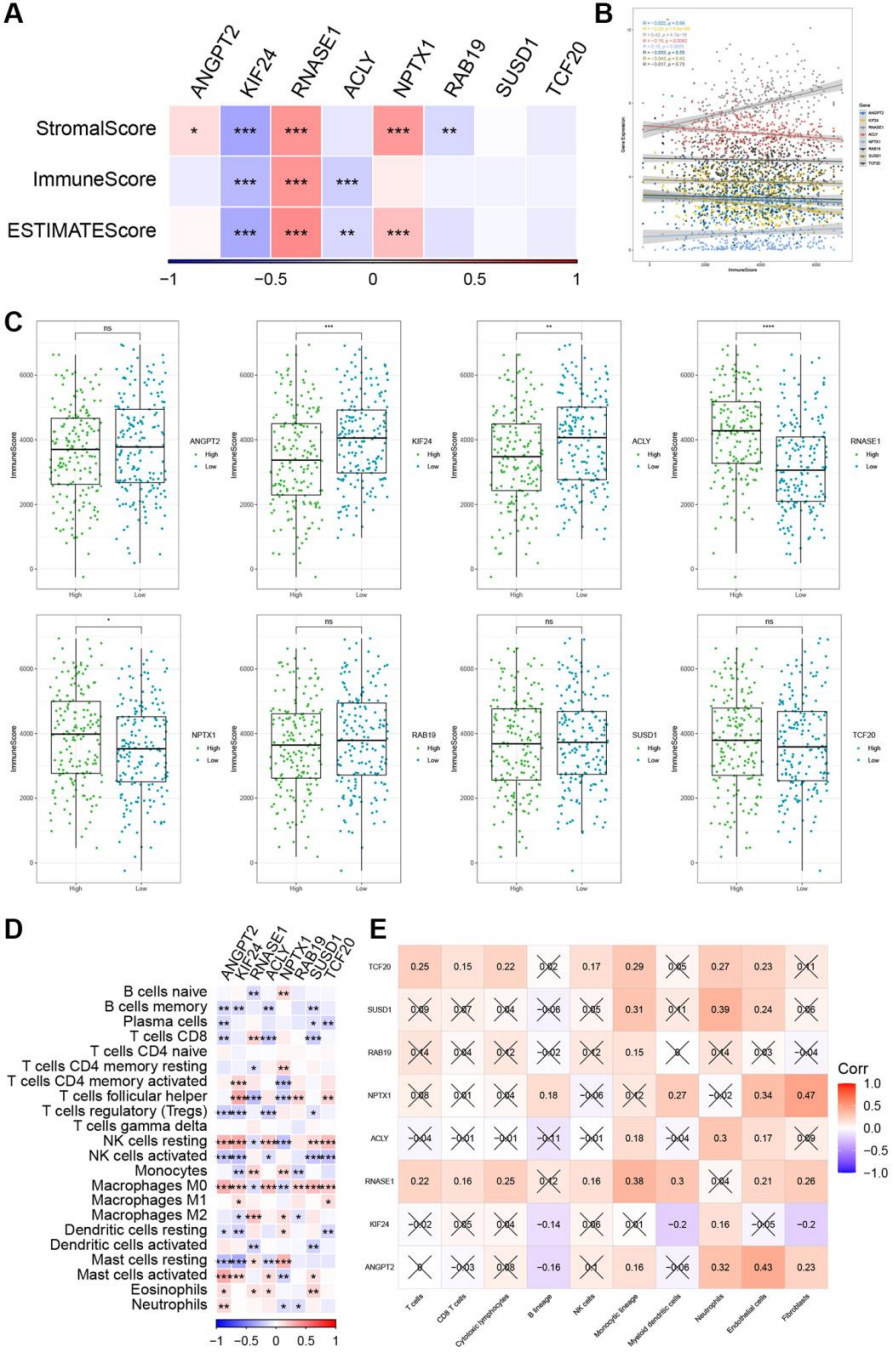
**The relationship between the risk genes and immune landscape.** (**A**, **B**) The correlation matrix of the risk genes and StromalScore, ImmuneScore, and ESTIMATEScore. (**C**) Comparison of high and low expression of 8 key genes and ImmuneScore. (**D**) Correlation between 8 key genes and immune cell score predicted by CIBERSORT analysis. (**E**) Correlation between 8 key genes and 10 immune cell types predicted by MCPcounter analysis. ^*^P < 0.05, ^**^P < 0.01, ^***^P < 0.001, and ^****^P < 0.0001.

Our data revealed significant positive correlations between RNASE1 and StromalScore, ImmuneScore, and ESTIMATEScore, whereas KIF24 exhibited significantly negative correlations with the three scores (Figure 9A, 9B). Based on the median of gene expression, we further evaluated the ImmuneScore difference between patients with high- and low-risk genes. The high-expression group for RNASE1 and NPTX1 genes exhibited a higher ImmunScore (Figure 9C). Correlation analysis reveals a significant negative correlation between ANGPT2, SUSD1, and ACLY with CD8 T cells, regulatory T cells, and activated NK cells, while ANGPT2, SUSD1, and ACLY show a significant positive correlation with resting NK cells, M0 macrophages, activated mast cells, and neutrophils (Figure 9D). Moreover, the MCPcounter algorithm revealed an association between ANGPT2 and neutrophils, endothelial cells, and fibroblasts (Figure 9E).

### Assessment of the predictive efficacy of risk models for immunotherapy

**Figure 10:**
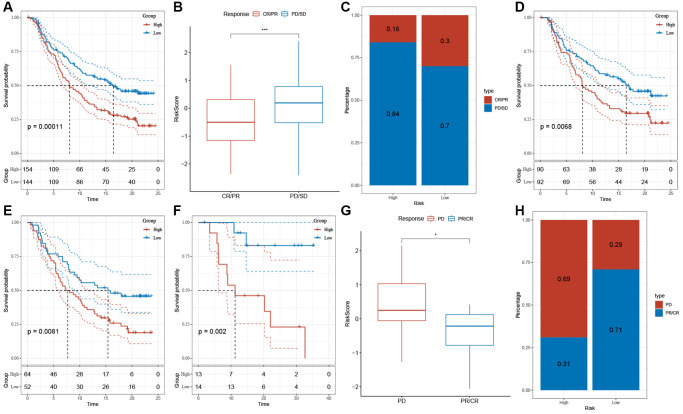
**The response of risk score to immune checkpoint inhibitors in IMvigor210 cohort and GSE78220 cohort.** (**A**) Prognostic differences among risk score groups in the IMvigor210 cohort. (**B**) Differences in risk scores among immunotherapy responses in the IMvigor210 cohort. (**C**) Distribution of immunotherapy responses among risk score groups in the IMvigor210 cohort. (**D**) Prognostic differences between risk score groups in early-stage patients in the IMvigor210 cohort. (**E**) Prognostic differences between risk score groups in advanced patients in the IMvigor210 cohort. (**F**) Prognostic differences in risk score groups in the GSE78220 cohort. (**G**) Differences in risk scores among immunotherapy responses in the IMvigor210 cohort. (**H**) Distribution of immunotherapy responses among risk score groups in the GSE78220 cohort. ^*^P < 0.05, ^***^P < 0.001.

Immunotherapy, represented by ICIs, has become a crucial therapeutic approach for extending the survival of patients with advanced tumors. The response of patients to immunotherapy determines the ultimate treatment benefits. Therefore, we evaluated the predictive efficacy of risk features for immune response based on the IMvigor210 and GSE78220 cohorts. In the IMvigor210 cohort, patients in the low-risk group showed a favorable prognosis and a prolonged OS compared to the high-risk group (Figure 10A, *p* = 0.00011). Similar patterns were observed in the GSE78220 cohort (Figure 10F, *p* = 0.002). Furthermore, in the IMvigor210 and GSE78220 datasets, the proportion of stable disease (SD) and progressive disease (PD) was higher in the high-risk group compared to the low-risk group (Figure 10B, 10C, 10G, 10H). Of note, there is a significant difference in survival between patients in Stages I+II (Figure 10D, *p* = 0.0068) and Stages III+IV (Figure 10E, *p* = 0.0081) in the high- and low-risk groups.

## DISCUSSION

The dynamic interplay among various components within the TME, including cancer cells, stromal cells, immune cells, and the extracellular matrix (ECM), plays a crucial role in fostering tumor heterogeneity, clonal evolution, and multidrug resistance mechanisms [35–38]. This process ultimately contributes to tumor progression and metastasis. It has been reported that proangiogenic factors secreted by TECs and their interaction with tumor and immune cells are vital for tumor proliferation, angiogenesis, metastasis, and chemoresistance [9, 16, 22, 39]. The main objective of this research is to examine the variety of TEC clusters to analyze and categorize TECs in GC. We discovered five TEC clusters exhibiting unique characteristics. Previous studies have confirmed that a specific TEC cluster is essential for GC development, implying worse clinical outcomes [40]. Our study identified five TEC clusters, four of which were significantly associated with the prognosis of GC. Notably, differences in cell cycle, HIPPO, NOTCH, and RAS among TEC clusters may participate in GC growth and metastasis.

The Hippo pathway is a critical tumor suppressor signaling pathway that maintains tissue homeostasis by regulating cell differentiation and proliferation [41–43]. The activation of YAP/TAZ in the Hippo signaling pathway greatly enhances cell proliferation, migration, invasion, and anti-apoptotic processes in different solid tumors, including GC [43–45]. It has been shown that DUB1, a deubiquitinating enzyme, shows significantly elevated expression in GC tissues and is correlated with the activated TAZ protein and patient prognosis [46]. Mechanistically, DUB1 inhibits TAZ K48-linked polyubiquitination, reducing TAZ degradation and increasing its stability, thereby promoting GC stemness and progression. Furthermore, the Hippo pathway interacts with other important oncogenic signaling pathways, such as Wnt, Notch, EGFR, PI3K/AKT, MAPK, etc., [47]. This interaction affects the key components of the Hippo pathway, thereby determining cell fate.

Based on the predictive values of three TEC clusters, we developed a risk signature consisting of 8 genes. It consisted of three protective genes (RAB19, KIF24, and TCF20) and five risk genes (ACLY, SUSD1, ANGPT2, RNASE1, and NPTX1). In our study, SNV mutations were observed in RAB19 and RNASE1 without significant co-occurrence probability. SNV mutations affect protein activity or function, leading to GC development. Notably, CDC27 and FLG genes were mutated at the single-cell level but not detected in the corresponding tumor tissues, and they could promote cell growth by modulating the inflammatory response. KLF4 is a tumor suppressor, and KIF4 SNV may affect its DNA binding ability and apoptotic function [48]. Research has discovered a prevalent SNV in the zinc finger 2 region of the KLF4 gene in the foveolar-type gastric adenoma (FGA) tissues of Helicobacter pylori-negative patients. Compared to the wild-type KLF4 gene, the mutated KLF4 significantly inhibits cell proliferation and induces early apoptosis [48].

Additionally, we discovered that the 8 genes exhibited a significant correlation with 6 pathways. The protective genes showed a strong positive correlation with MITOTIC SPINDLE, E2F targets, G2M checkpoints, and spermatogenesis, while the risk genes (such as KIF24 and NPTX1) were significantly linked to myogenesis and UV-response-DN. E2F-1 participates in the G2/M checkpoint process, and as a transcription factor, it regulates the cell cycle, proliferation, and apoptosis. Overexpression of E2F-1 can decrease the expression of c-Myc, Skp2, Bcl-2, cyclin D1, and survivin, while increasing the expression of Bax, ultimately inhibiting the growth of GC cells [49, 50]. Furthermore, G2/M checkpoint arrest significantly inhibits the occurrence and development of GC [51, 52]. Hence, this data offers guidance for further investigating the regulatory mechanism of these risk genes in GC.

Recent evidence suggests that the interplay between TECs and the tumor immune microenvironment (TIME) plays a crucial role in tumor progression [9, 22]. Tumor-associated lymphatic endothelial cells (LECs) promote the growth and remodeling of lymphatic vessels by responding to factors in the TME, increasing lymph node metastasis [53]. Our research discovered a positive correlation between RNASE1 and ImmuneScore and a negative correlation between KIF24, ACLY, and ImmuneScore. These results imply potential crosstalk between risk genes and the TME in GC, highlighting their potential as therapeutic targets.

In the TME, various immune cell populations collectively determine the anti-tumor immune status of GC patients [35, 54, 55]. TECs can interact with these immune cells, establishing an immunosuppressive TME that facilitates tumor cell evasion from immune surveillance [8, 14]. In the risk model, memory B cells, plasma cells, regulatory T cells, activated NK cells, and activated dendritic cells show negative correlation with risk genes. Furthermore, our data suggest that a risk signature based on TECs can predict responses to immunotherapy. These findings provide new insights into the biological role of TECs in tumor immunity. However, the molecular mechanisms of TECs in GC and their potential value in immunotherapy still require further exploration.

Despite our progress in understanding the role of TECs in the development of GC, there remain some limitations. Firstly, the TEC clusters and the TEC-based risk signature we constructed are based on retrospective data from public databases, meaning our conclusions could be limited by the data collection and analysis methodologies. To ensure the accuracy and reliability of these findings, future research should be validated in a prospective, multicentric cohort of GC patients to better simulate the situation in clinical practice and provide stronger evidence to support our conclusions. Secondly, our study mainly focused on exploring the potential prognostic value of the TEC-based risk signature, that is, predicting the prognosis of GC patients by analyzing TEC characteristics. However, we have not fully revealed the specific molecular mechanisms involved by TECs in the occurrence and development of GC. To deeply understand how TECs affect the biological process of GC, future research needs to adopt a variety of experimental methods, including gene expression analysis, proteomics studies, and experiments using cell and animal models, to reveal the molecular action pathways of TECs in GC and potential therapeutic targets. Moreover, we also need to consider the heterogeneity of GC, that is, different types of GC patients may have different TEC characteristics and risk patterns. Therefore, future research should consider a more detailed classification of GC subtypes and explore the role of TECs in different subtypes, in order to provide more personalized treatment plans for patients.

Finally, although our research provides a new perspective for the prognosis assessment and treatment of GC, we must recognize that any single biomarker or risk signature cannot completely predict the progression of the disease. Future studies should explore integrating multiple biomarkers with clinical parameters to develop a more comprehensive prognostic model for risk assessment, enhancing the accuracy and effectiveness of GC treatment.

## CONCLUSION

Our study delineated five distinct TEC clusters within GC tissues, four of which exhibit significant associations with the prognosis of GC patients. Utilizing lasso-Cox analysis, we identified 8 risk genes to formulate a TEC-based risk signature, which holds high clinical application value by accurately predicting the prognosis of GC patients and their response to immunotherapy.

## Supplementary Materials

Supplementary Figures
