# Supplementary material for: Characterization of tumor endothelial cells (TEC) in gastric cancer and development of a TEC-based risk signature using single-cell RNA-seq and bulk RNA-seq data
Source: Aging (Albany NY). 2024 Jun 12;16(12):10252–70. doi: 10.18632/aging.205928 (PMC11236301; doi:10.18632/aging.205928)
Supplement: Supplementary Figures [file aging-16-205928-s001.pdf]

SUPPLEMENTARY FIGURES

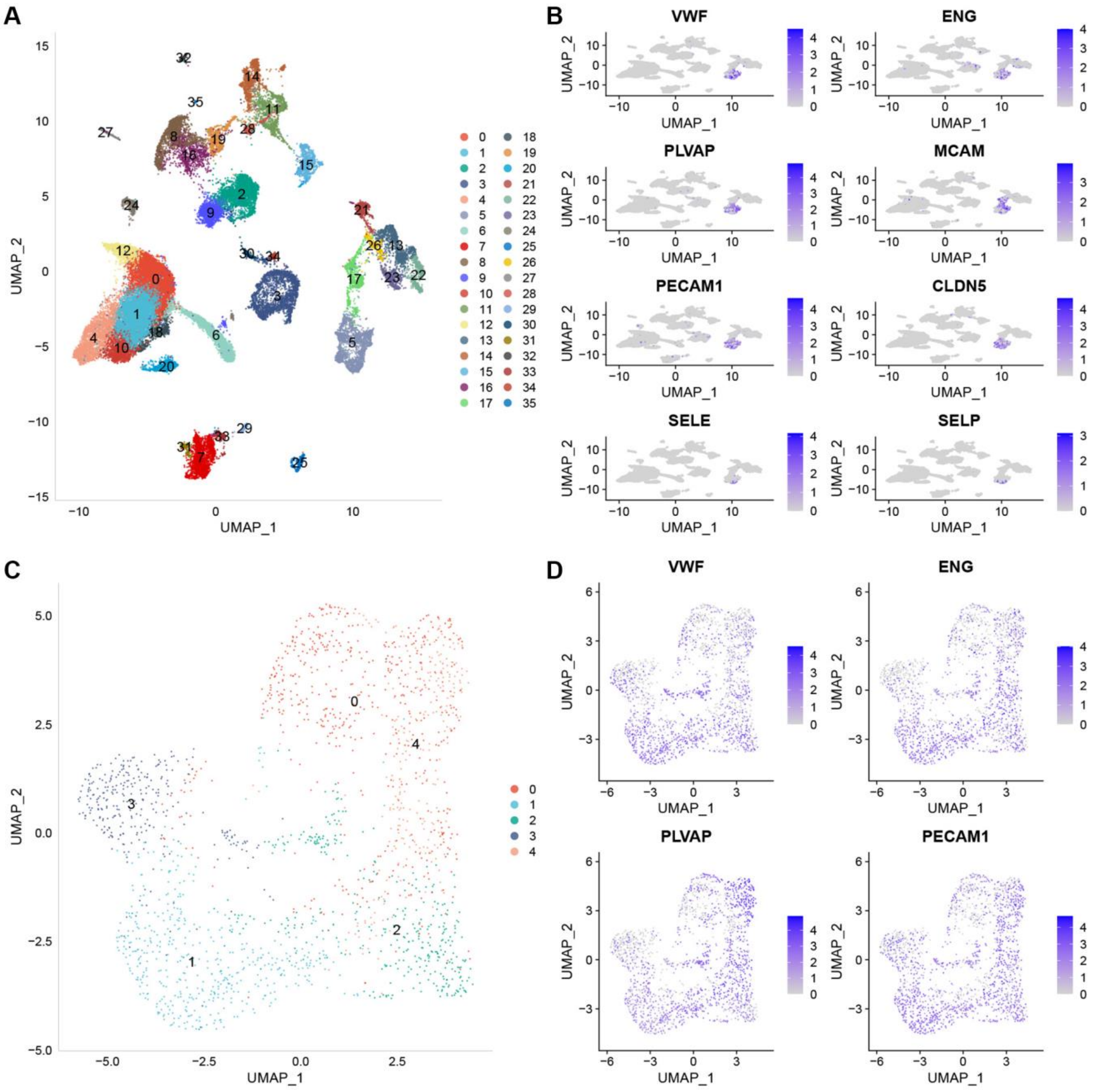

Supplementary Figure 1. The clustering of TEC populations and dimensionality reduction. (**A**) Distribution of subpopulations after clustering of all cells. (**B**) UMAP map of TEC marker gene expression. (**C**) Distribution of subpopulations after re-clustering of TEC. (**D**) UAMP diagram of marker expression in five TEC clusters.

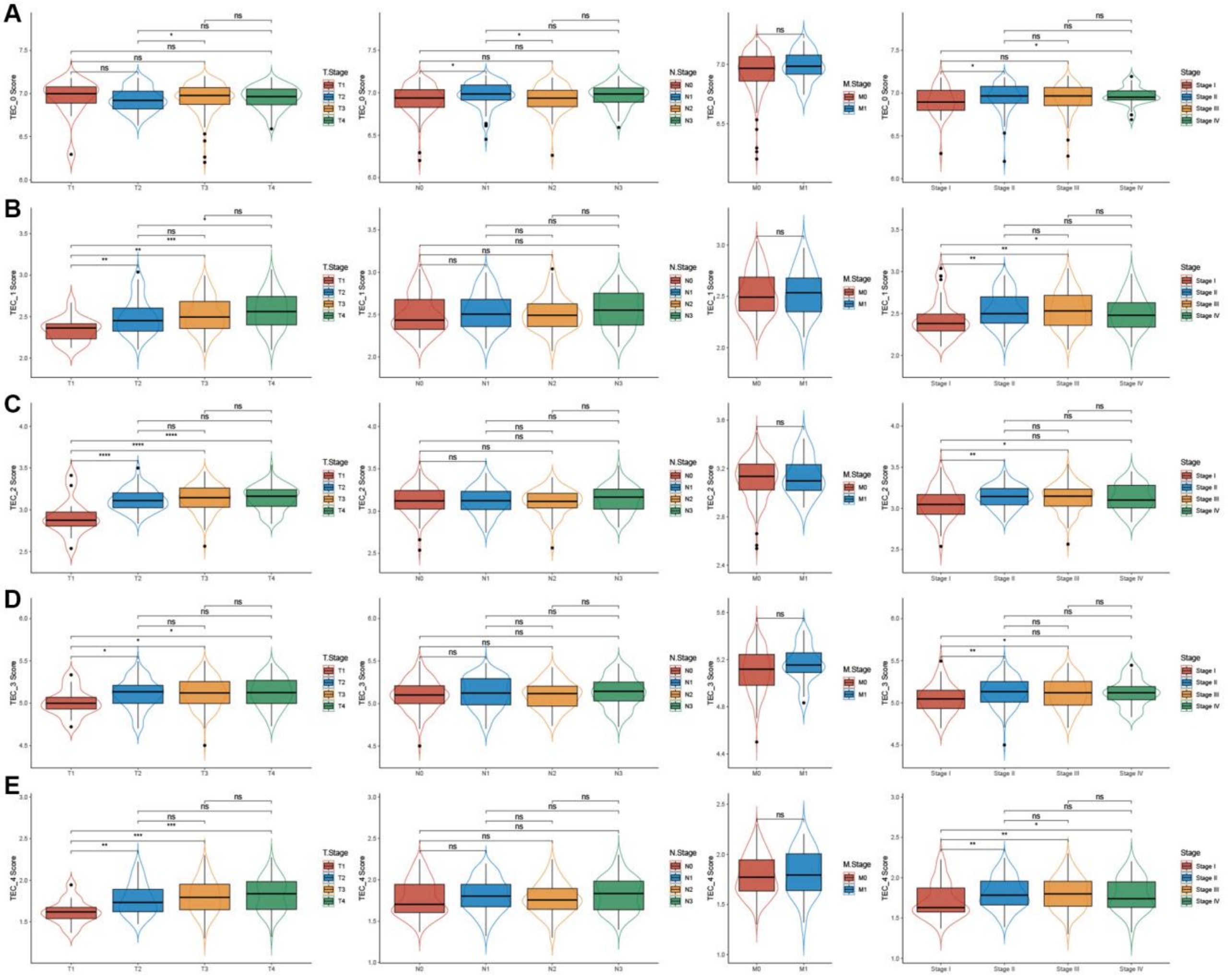

Supplementary Figure 2. The differences between TEC scores and clinical variables in five TEC clusters (**A**–**E**).
